# CNS progenitor cells and oligodendrocytes are targets of chemotherapeutic agents *in vitro *and *in vivo*

**DOI:** 10.1186/jbiol50

**Published:** 2006-11-30

**Authors:** Joerg Dietrich, Ruolan Han, Yin Yang, Margot Mayer-Pröschel, Mark Noble

**Affiliations:** 1 Department of Biomedical Genetics, University of Rochester Medical Center, 601 Elmwood Avenue, Rochester, NY 14642, USA

## Abstract

**Background:**

Chemotherapy in cancer patients can be associated with serious short- and long-term adverse neurological effects, such as leukoencephalopathy and cognitive impairment, even when therapy is delivered systemically. The underlying cellular basis for these adverse effects is poorly understood.

**Results:**

We found that three mainstream chemotherapeutic agents – carmustine (BCNU), cisplatin, and cytosine arabinoside (cytarabine), representing two DNA cross-linking agents and an antimetabolite, respectively – applied at clinically relevant exposure levels to cultured cells are more toxic for the progenitor cells of the CNS and for nondividing oligodendrocytes than they are for multiple cancer cell lines. Enhancement of cell death and suppression of cell division were seen *in vitro *and *in vivo*. When administered systemically in mice, these chemotherapeutic agents were associated with increased cell death and decreased cell division in the subventricular zone, in the dentate gyrus of the hippocampus and in the corpus callosum of the CNS. In some cases, cell division was reduced, and cell death increased, for weeks after drug administration ended.

**Conclusion:**

Identifying neural populations at risk during any cancer treatment is of great importance in developing means of reducing neurotoxicity and preserving quality of life in long-term survivors. Thus, as well as providing possible explanations for the adverse neurological effects of systemic chemotherapy, the strong correlations between our *in vitro *and *in vivo *analyses indicate that the same approaches we used to identify the reported toxicities can also provide rapid *in vitro *screens for analyzing new therapies and discovering means of achieving selective protection or targeted killing.

## Background

One of the disturbing findings to emerge from studies on cancer survivors is the frequency with which chemotherapy is associated with adverse neurological sequelae. Adverse neurological effects associated with treatment of both childhood and adult cancers range from abnormalities detected by CNS imaging (for example, damage to white matter) [1-3] to clinical symptoms. Neurological complications observed as a consequence of chemotherapy include leukoencephalopathy, seizures, cerebral infarctions, and cognitive impairment [4-10].

While it is perhaps not surprising that neurotoxicity occurs after localized delivery of chemotherapeutic agents to the CNS, it is increasingly apparent that this is also a substantial problem associated with the systemic delivery of these agents for treatment of non-CNS tumors [11-18]. For example, current data suggest that 18% of all breast cancer patients receiving standard-dose chemotherapy show cognitive defects on post-treatment evaluation [19], and such problems were reported in more than 30% of patients examined two years after treatment with high-dose chemotherapy [7,8], a greater than eightfold increase over the frequency of such changes in control individuals. Even these numbers may be underestimates of the frequency of adverse neurological sequelae in association with aggressive chemotherapy, as two longitudinal studies on breast cancer patients treated with high-dose chemotherapy with carmustine (BCNU), cisplatin, and cyclophosphamide, and evaluated using magnetic resonance imaging and proton spectroscopy, have shown that changes in white matter in the CNS induced by the treatment could occur in up to 70% of individuals, usually with a delayed onset of several months after treatment [1,2]. Even if examination of all cancers were to lower the frequency of these problems to 25% of the lower estimates (that is, around 4.5% of patients receiving low-dose therapy and 7.5% of patients receiving high-dose chemotherapy) the prevalence of cancer in the world's populations means that the total number of individuals for whom adverse neurological changes are associated with cancer treatment is as great as for many of the more widely recognized neurological syndromes.

Despite the clear evidence of the neurotoxicity of at least some forms of chemotherapy, studies on the effects of chemotherapeutic compounds on brain cells are surprisingly rare. For example, it is known that application of methotrexate directly into the ventricles of the brain is associated with ventricular dilation, edema, and the visible destruction of the ependymal cell layer lining the ventricles and the surrounding brain tissue [20]. Application of cytosine arabinoside (cytarabine) onto the surface of the brain is also associated with adverse effects on the dividing cells of the subventricular zone of the CNS [21]. *In vitro *studies [22] have also shown that oligodendrocytes are vulnerable to killing by carmustine (BCNU, an alkylating agent used in the treatment of brain tumors, myeloma, and both Hodgkin and non-Hodgkin lymphoma) at doses that would be routinely achieved during treatment. In general, however, relatively little is known about the effects of chemotherapeutic agents on the cells of the CNS, in striking contrast to the extensive investigations on the effects of irradiation on the brain.

To investigate the biological basis of the adverse neurological consequences of chemotherapy, we posed the following questions. Which cells are vulnerable? Is vulnerability restricted to dividing cells? Does toxicity reflect a direct action of chemotherapeutic agents on defined neural populations? How does the sensitivity of primary neural cells compare with that of cancer cells? What are the *in vivo *effects of chemotherapy on the dividing populations of the CNS? Do chemotherapeutic agents with different modes of action target the same or different populations of normal cells?

## Results

### Neural progenitor cells are more vulnerable to DNA cross-linking agents *in vitro *than are many cancer cell lines

**Figure 1:**
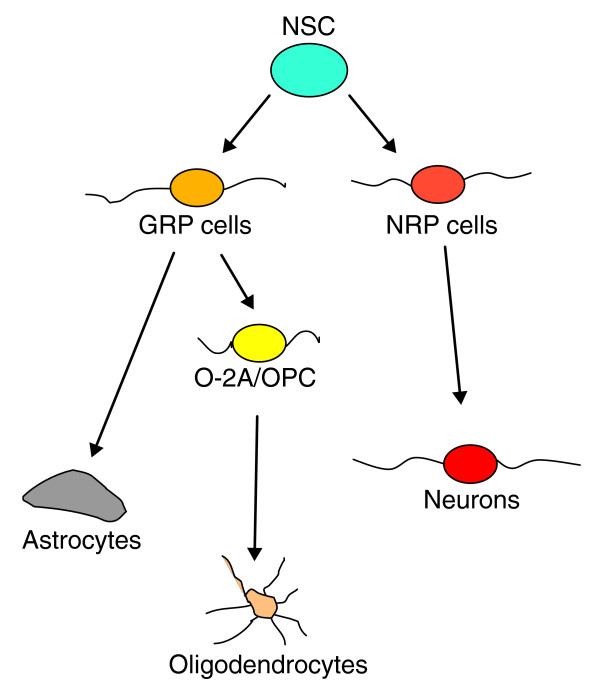
Schematic representation of the lineage relationships of the cell types examined in these studies. Pluripotent neuroepithelial stem cells (NSC) give rise to glial-restricted precursor (GRP) cells and neuron-restricted precursor (NRP) cells. NRP cells can give rise to multiple populations of neurons, whereas GRP cells give rise to astrocytes and oligodendrocyte-type-2 astrocytes (O-2A/OPCs). The O-2A/OPCs in turn give rise to oligodendrocytes. The progenitor cells that lie between NSCs and differentiated cell types, and are the major dividing cell population in the CNS, appear to be exceptionally vulnerable to the effects of chemotherapeutic agents. Also sharing this vulnerability are nondividing oligodendrocytes.

To determine the sensitivity of CNS cells to chemotherapeutic agents, we first exposed a large variety of cell types to BCNU and cisplatin, of which the former is primarily used for treating brain cancers and Hodgkin's lymphoma and the latter is used to treat a wide range of cancers (including breast, lung and colon cancers, multiple myeloma and Hodgkin's lymphoma). Both agents have been associated with significant CNS toxicity in patients [11,23-25]. Cisplatin is an alkylating agent thought to work primarily through forming intrastrand crosslinks between adjacent purine bases [26], whereas the nitrosourea BCNU causes primarily interstrand crosslinking between guanine and cytosine [27]. To ensure that we analyzed the direct effects of these compounds on potential target cells, we applied BCNU or cisplatin to purified populations of neuroepithelial stem cells (NSCs, which generate all neural cells of the CNS [28]), neuron-restricted precursor (NRP) cells (which generate neurons but not glia [29]), glial-restricted precursor (GRP) cells (which generate the macroglia of the CNS but not neurons [30]), and oligodendrocyte-type-2 astrocyte (O-2A) progenitor cells (also referred to as oligodendrocyte precursor cells, and here abbreviated as O-2A/OPCs, the direct ancestors of oligodendrocytes [31]), astrocytes, and oligodendrocytes (the myelin-forming cells of the CNS) (all summarized in Figure 1). We also analyzed human NSCs and GRP cells [32] and human tumor cell lines from uterine (MES), breast (MCF-7), colon (HT-29, SW-480) and ovarian (ES-2) cancers, a meningioma cell line and several glioma cell lines (1789, T98, UT-12, UT-4). Methodological information is given in the Materials and methods.

**Figure 2:**
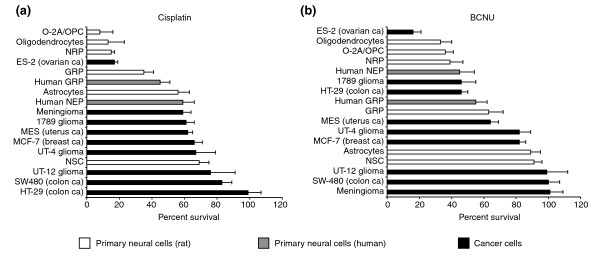
Primary CNS cells are more vulnerable to BCNU and cisplatin than are cancer cells. Cells were plated on coverslips in 24-well plates at a density of 1,000 cells per well and allowed to grow for 24–48 h. On the basis of drug concentrations achieved in human patients, cells were exposed to **(a) **cisplatin (1 μM; for 20 h) or **(b) **BCNU (25 μM; for 1 h). Cell survival and viability was determined after additional 24–48 h (see Materials and methods). The rat neural cell types studied included O-2A/OPCs, oligodendrocytes, NRP cells, GRP cells, NSCs, and astrocytes. The normal human neural cell types consisted of human GRP and neuroepithelial precursor cells (human NEP). The tumor cells studied were the human malignant glioma cells UT-4, UT-12, and 1789, the colon cancer cell lines HT-29 and SW480, a meningioma cell line (Men-1), breast cancer cells (MCF-7), uterine cancer cells (MES), and ovarian cancer cells (ES-2). Each experiment was carried out in quadruplicate and repeated multiple times in independent experiments. Data represents mean of survival ± SEM, normalized to control values.

Clinically relevant concentrations of BCNU or cisplatin were more toxic for lineage-committed progenitor cells and for NSCs than they were for cancer cells. For example, exposure to 1 μM cisplatin or 25 μM BCNU caused 60–90% reductions in the viability of O-2A/OPCs and NRP cells (Figure 2), but had little effect on most of the cancer cell lines examined. The toxicity of cisplatin was extensive even at concentrations as low as 0.1 μM, killing 40% or more of the populations of O-2A/OPCs, oligodendrocytes, and NRP cells at this low exposure. Exposure to 5 μM BCNU was also toxic for O-2A/OPCs, NRP cells, and oligodendrocytes. Thus, these sensitivities were observed at exposure levels corresponding to the low range of concentrations in cerebrospinal fluid (CSF) associated with cancer treatment. These are as low as 0.6–2.8 μM for low-dose intravenous applications of cisplatin [33] and 8–10 μM for similar applications of BCNU [34,35], and can be up to two orders of magnitude or greater in high-dose applications [36-40].

**Figure 3:**
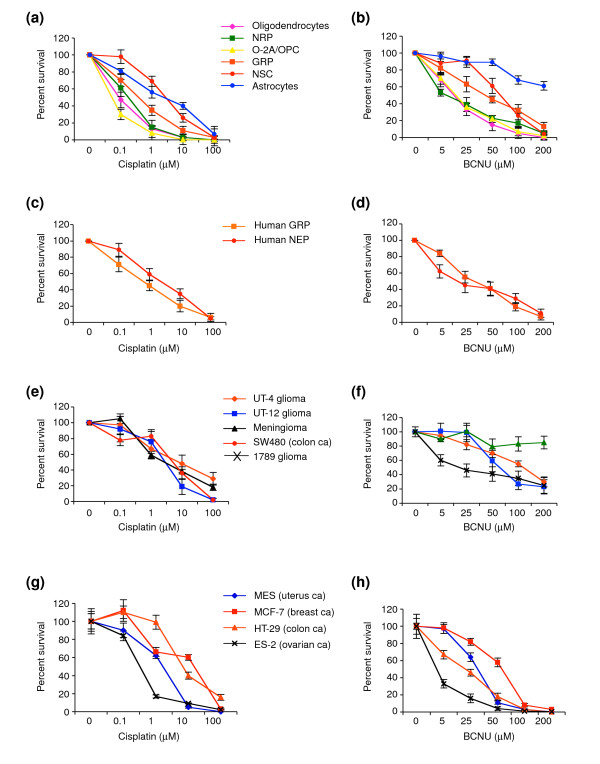
Sensitivity of rat and human-derived CNS cells and human cancer cells to BCNU or cisplatin. Cells were treated with **(a,c,e,g) **cisplatin and **(b,d,f,h) **BCNU over a wide dose range (0.1–100 μM and 5–200 μM, respectively). Each experiment was carried out in quadruplicate and repeated multiple times in independent experiments. Data represents mean of survival ± SEM, normalized to control values. There are no concentrations of either drug for which tumor cell lines were more sensitive than the more sensitive neural progenitor cells and oligodendrocytes.

Increasing cisplatin or BCNU concentrations to levels that killed 40–80% of cancer cells caused 70–100% reduction in viability of O-2A/OPCs, GRP cells, NRP cells, and NSCs (Figure 3). The preferential vulnerability of both rat and human primary CNS progenitor cells to BCNU and cisplatin was apparent also at very low exposure levels. Even the BCNU- and cisplatin-responsive ES-2 ovarian cancer cell line was only as vulnerable as normal CNS progenitors. Thus, in examining tumors of a wide range of sensitivities, we could not identify any populations that exceeded the vulnerability of neural precursor cells to damage induced by cisplatin or BCNU.

One of the unexpected findings to emerge from our studies was that the vulnerability of CNS cells to BCNU and cisplatin was not restricted to rapidly dividing cells, as nondividing oligodendrocytes were as sensitive as neural progenitors to BCNU and cisplatin, consistent with our previous studies on vulnerability of oligodendrocytes to BCNU [22]. Thus, contrary to the widely held view that the toxicity of chemotherapeutic agents is primarily directed against dividing cells, the ability of BCNU and cisplatin to damage normal cell types in the CNS was not limited to rapidly dividing progenitors. Moreover, cell division by itself was not sufficient to confer vulnerability, as rapidly dividing NSCs were more resistant than progenitor cells. Of all the CNS cell types examined, only astrocytes were as resistant as cancer cells. Thus, the major targets of cisplatin and BCNU toxicity appear to be lineage-restricted progenitor cells and nondividing oligodendrocytes.

### Sub-lethal doses of chemotherapy reduce the self-renewal of O-2A/OPCs

Normal progenitor cell function also requires cell division, both during development and for purposes of repair. For O-2A/OPCs, where division can be followed over several days in sensitive clonal assays, it is known that agents that can be cytotoxic at high concentrations will induce cessation of division and induction of differentiation when applied at sub-lethal dosages [41]. We therefore asked whether sub-lethal concentrations of cisplatin and BCNU compromised progenitor cell proliferation. These assays were conducted on O-2A/OPCs in order to benefit from the ability to examine proliferation and differentation at the clonal level [41-43].

**Figure 4:**
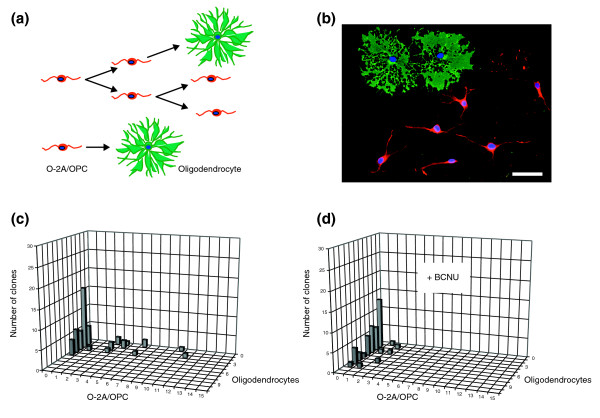
A low dose of BCNU decreases division and promotes differentiation of O-2A/OPCs. Cells grown at clonal density were exposed 1 day after plating to low-dose BCNU (2.5 μM for 1 h), a dosage that did not cause significant killing (< 5%) of O-2A/OPCs in mass culture. The number of undifferentiated O-2A/OPCs and differentiated cells (oligodendrocytes) was determined in each individual clone from a total number of 50 clones in each condition by morphological examination and by immunostaining with A2B5 and anti-GalC (galactocerebroside) antibodies (to label O-2A/OPCs and oligodendrocytes, respectively). **(a) **Schematic diagram of the differentiation potential of O-2A/OPCs. Bipolar O-2A/OPCs can undergo continued cell division(s) to form new precursor cells (red), and can differentiate into multipolar postmitotic oligodendrocytes (green). Alternatively, an O-2A/OPC can differentiate directly into an oligodendrocyte without further cell divisions. **(b) **An example of one clone in culture. Immunostaining with A2B5 (red) and anti-GalC (green) identifies six O-2A/OPCs and two oligodendrocytes. Cell nuclei stained in blue (DAPI). Scale bar represents 20 μm. **(c) **Composition of progenitors and oligodendrocytes in a representative experiment of control cultures analyzed 8 days after plating optic nerve-derived O-2A/OPCs at clonal density. Multiple clones with three or more O-2A/OPCs were seen. **(d) **In parallel BCNU-treated cultures, analyzed 8 days after plating at clonal density (7 days after BCNU exposure), no clones contained more than two O-2A/OPCs. Experiments were performed in triplicate in at least two independent experiments. In the experiments represented in (c) and (d) the proliferation and differentiation of O-2A/OPCs were followed over a time course of up to 10 days after BCNU treatment. Results are presented as representative three-dimensional graphs. The number of progenitors per clone is shown on the *x *(horizontal) axis, the number of oligodendrocytes on the *z *(orthogonal) axis and the number of clones with any particular composition on the *y *(vertical) axis.

Transient exposure of O-2A/OPCs to concentrations of cisplatin or BCNU that did not cause significant cell death (0.05 μM cisplatin or 2.5 μM BCNU) was associated with reduced cell division and increased differentiation into oligodendrocytes (Figure 4). In control cultures, for example, 35% of the cells were dividing progenitors after seven days, and more than 25% of clones contained three or more progenitors. In striking contrast, in cultures exposed to 2.5 μM BCNU for just 1 hour after the first day of *in vitro *growth and then followed for an additional seven days, only 6% of cells were progenitors and no clones contained more than two progenitor cells. Similar observations were seen at earlier time points and also with transient application of cisplatin to O-2A/OPCs (data not shown). Thus, even when cell death is not evident, these agents may compromise progenitor cell division. As average clonal sizes in the BCNU-exposed versus control cultures at day 7 were not significantly different (3.3 ± 2.3 vs 3.6 ± 2.3 cells per clone in BCNU-treated vs control cultures, respectively; *P *= 0.55), it seems that the very low concentration of BCNU examined in these studies is sufficient to shift the balance between division and differentiation far enough in the direction of oligodendrocyte generation to have a cumulative effect over multiple cellular generations, but not to immediately cause cell-cycle exit. As considered in the Discussion, these results are much like those seen in our ongoing studies on the regulation of the balance between division and differentiation by intracellular redox state and by signaling molecules that make O-2A/OPCs more oxidized. The possibility that this effect of exposure to very low concentrations of BCNU (along with cisplatin and, as shown later, cytarabine) is related to oxidative changes is considered in the Discussion.

### *In vivo *effects of BCNU and cisplatin on cell death and cell division in the CNS

**Figure 5:**
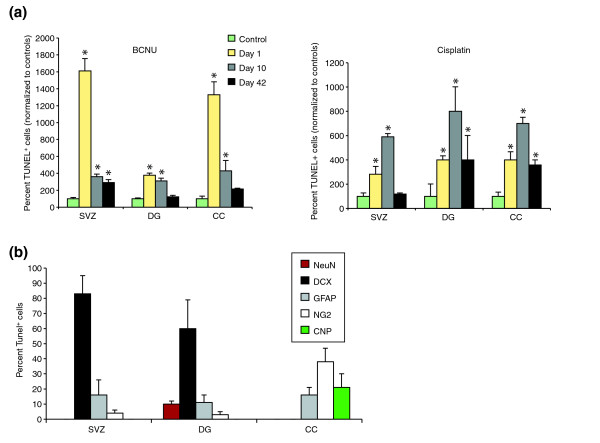
Systemic chemotherapy leads to increased and prolonged cell death in the adult mouse CNS. Cell death was determined using the terminal deoxynucleotidyltransferase-mediated dUTP nick-end labeling (TUNEL) assay. The number of TUNEL^+ ^cells was analyzed in control animals (which received 0.9% NaCl i.p.) and chemotherapy-treated animals and presented as percent normalized values of controls. Each treatment group consisted of *n *= 5 animals, including control groups at each time point. **(a) **Animals that received three BCNU (left panel) or cisplatin (right panel) injections (10 mg/kg or 5 mg/kg, respectively, on days 1, 3, and 5) show marked and prolonged increases in cell death in the lateral subventricular zone (SVZ), the corpus callosum (CC) and the dentate gyrus (DG) at 1, 10, and 42 days following treatment (*n *= 5 animals per group). *P < 0.01. **(b) **Co-analysis of TUNEL labeling with antigen expression reveals that the great majority of TUNEL^+ ^cells in the SVZ and DG are doublecortin^+ ^(DCX^+^) neuronal progenitors [44], and that other TUNEL^+ ^cells include GFAP^+ ^cells (which may be stem cells or astrocytes [45]) and NG2^+ ^progenitor cells [46]. In the CC, in contrast, the TUNEL^+ ^cells were NG2^+ ^glial progenitor cells [47], CNPase^+ ^(CNP^+^) oligodendrocytes or GFAP^+ ^astrocytes. Co-labeling for TUNEL and myelin basic protein expression revealed results similar to CNPase analysis. Note that close to 100% of TUNEL^+ ^cells are accounted for by known lineage markers.

Analyses *in vivo *confirmed that precursor cells and oligodendrocytes were also adversely affected by chemotherapeutic agents when systemically applied to living animals, and that these adverse effects continued beyond the period of chemotherapy exposure. In these experiments we treated mice with BCNU or cisplatin and examined cell death and cell division in the CNS. Treatment with three injections of BCNU (10 mg/kg each, given intraperitoneally (i.p.) on days 1, 3, and 5) was associated with significantly increased cell death for at least 6 weeks after treatment (Figure 5). Analysis using the terminal deoxynucleotidyltransferase-mediated dUTP nick-end labeling (TUNEL) assay for apoptotic cells (see Materials and methods) 1 day after completion of treatment revealed a 16.1-fold increase in the number of TUNEL^+ ^cells in the subventricular zone (SVZ), a 13.3-fold increase in the corpus callosum (CC), and a 3.8-fold increase in the dentate gyrus (DG) of the hippocampus. Ten days after the last injection there were still increased numbers of TUNEL^+ ^cells in all regions examined, and this increase was maintained in the SVZ for at least 6 weeks post-treatment (*P *< 0.04). Thus, application of BCNU was associated with the induction of an extended period of increased cell death.

Cisplatin (5 mg/kg i.p., days 1, 3, and 5) was similar to BCNU in its effects on the DG, and was associated with a prolonged two- to threefold increase in the number of TUNEL^+ ^cells, persisting at least 42 days, compared with sham-injected control animals. In contrast to BCNU, however, cisplatin was associated with only a modest increase in the number of TUNEL^+ ^cells in the CC at 10 days post-treatment (Figure 5), and with no significant increases in apoptotic cells in the SVZ.

To determine whether acute treatment with chemotherapy has the same cellular targets *in vivo *as *in vitro*, we combined the TUNEL assay with labeling with cell-type specific antibodies, and analyzed individual cells by confocal microscopy. In order to focus on the immediate targets of the chemotherapy, analysis was conducted in animals sacrificed 1 day after the completion of BCNU treatment.

**Figure 6:**
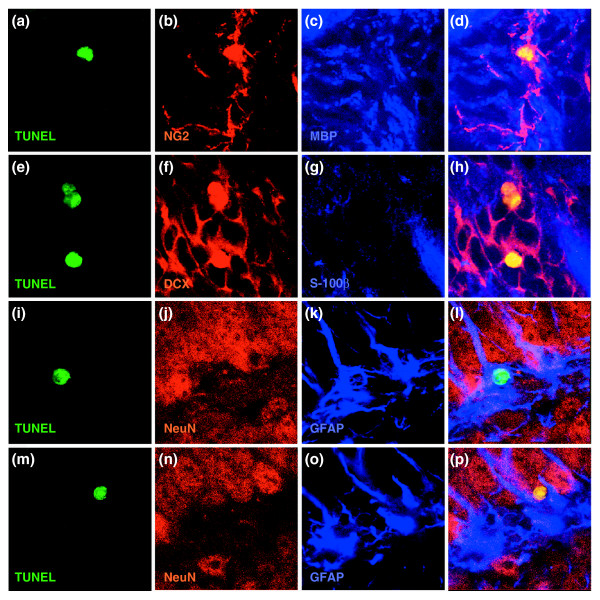
Representative images of co-labeling for TUNEL and expression of cell type-specific antigens. Despite the apparent labeling of nuclei with cell-type specific antibodies in dying cells (presumably due to the changes in antigen distribution associated with nuclear fragmentation), co-labeling was highly cell-type specific (see also Figure 7 for *z*-stack analysis). **(a-d) **NG2^+^/TUNEL^+ ^cells from the CC. In this and subsequent rows, the first image is of TUNEL staining, the next two images are of staining for the proteins indicated, and the merged image is on the far right. **(e-h) **DCX^+^/TUNEL^+ ^cells from SVZ; **(i-l) **GFAP^+^/TUNEL^+ ^cell from DG. **(m-p) **NeuN^+^/TUNEL^+ ^cell from DG. In all merged images except (l) co-labeled cells show up as yellow; in (l) the nucleus of the co-labeled cell is green. Magnification 400×.

**Figure 7:**
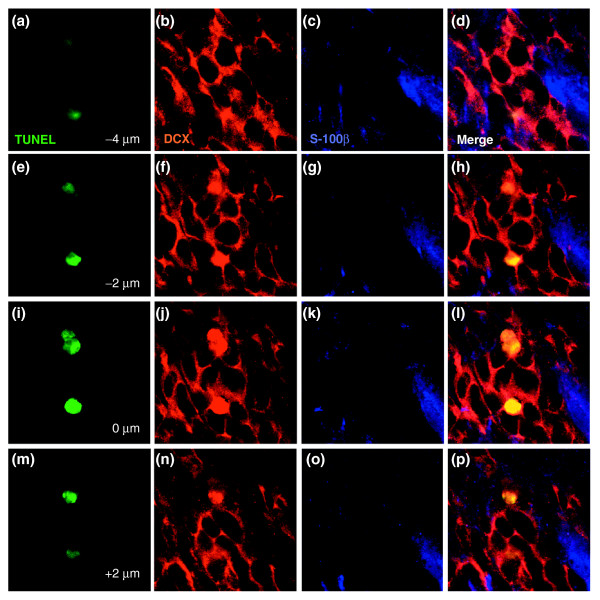
Representative *z*-stack of TUNEL^+^/doublecortin^+ ^cells. Photographs were taken at 2 μm intervals. Identical analyses were conducted for every cell that was scored as TUNEL^+ ^and expressing a cell type-specific antigen, as shown in Figure 4. Each row shows, from left to right, TUNEL staining, doublecortin staining, S-100b staining, and the merged image. **(a-d) **Images taken at -4 μm; **(e-h) **-2 μm; **(i-l) **0 μm; **(m-p) **2 μm. The congruence between the doublecortin^+ ^staining and the TUNEL^+ ^nuclei (which shows up as yellow in the merged image) was presumably due to the changes in antigen distribution associated with nuclear fragmentation, as this was always cell-type specific in that there was overlap only in those cases in which the rest of the cell was also stained with the same antibody. For example TUNEL^+^/doublecortin^+ ^cells were always doublecortin^+ ^in the cytoplasm, and other antibodies used in the same sections did not label the TUNEL-labeled nuclei of doublecortin^+ ^cells.

Confocal microscopic analysis of immunolabeling and TUNEL staining confirmed the vulnerability of precursor cells and oligodendrocytes *in vivo *(Figures 5, 6, 7). Untreated animals harbored only very rare TUNEL^+ ^cells, but such cells were frequently found in the SVZ, DG, and CC of animals receiving chemotherapy. In the SVZ and DG, the majority of TUNEL^+ ^cells observed after BCNU treatment were neuronal progenitors positive for the protein doublecortin (DCX) [44], followed by cells positive for glial fibrillary acidic protein (GFAP) (which may be astrocytes or stem cells [45]). We also observed co-labeling of a smaller number of TUNEL^+ ^progenitor cells also positive for the protein NG2 proteoglycan (which would be O-2A/OPCs [46,47]) and, in the DG, mature neurons positive for neuronal nuclear anitgen (NeuN). In the CC, most TUNEL^+ ^cells were NG2^+ ^glial progenitors, followed by oligodendrocytes (recognized by expression of 2',3'-cyclic nucleotide-3'-phosphodiesterase (CNPase) or myelin basic protein) and then by GFAP^+ ^cells (which were most probably astrocytes). In all tissues, labeling with these lineage markers accounted for the great majority of all TUNEL^+ ^cells. Thus, the profile of vulnerability agrees closely with that indicated by *in vitro *experiments, with sensitivity to the chemotherapeutic agents seen in both neuronal and glial progenitor cells, as well as in oligodendrocytes themselves.

We also examined the incorporation of bromodeoxyuridine (BrdU) into regions of the CNS in which cell division occurs in adult animals. Such division is highly restricted in the adult CNS, occurring only in particular regions and/or cell types. The SVZ is known to contain dividing cells and represents the major germinal zone in the CNS [48-51]. The hippocampus is also a region of continued cell generation in the adult CNS, with the majority of dividing cells appearing to be neuronal precursor cells [52,53]. White matter tracts also contain dividing cells that have been characterized as an adult-specific population of O-2A/OPCs. Although *in vitro *studies have shown that such cells may have long cell-cycle times, dividing *in vitro *over an average period of 65 hours instead of the 18-hour cell cycle displayed by O-2A/OPCs isolated from young postnatal rats [54,55], their frequency in the adult CNS is such that they actually appear to be the major dividing cell type in this tissue [56,57].

**Figure 8:**
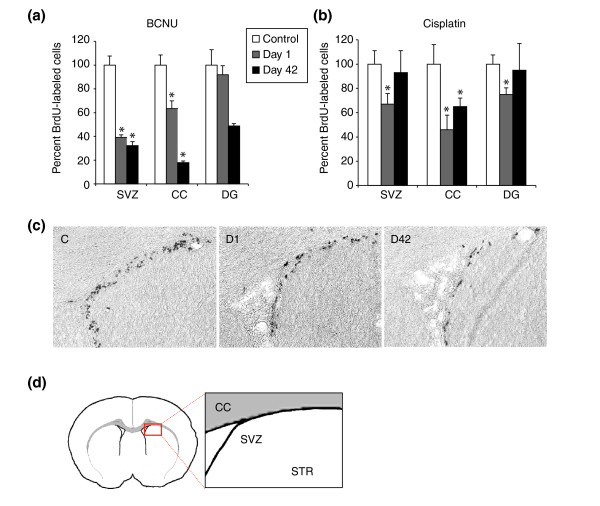
Chemotherapy decreases cell proliferation in the adult mouse CNS. Systemic exposure to cisplatin and BCNU was associated with profound changes in the number of BrdU-incorporating cells in the lateral SVZ, the DG and the CC. Animals were treated as described in Figure 5. The graphs show the percent-corrected values of BrdU^+ ^cells per brain area normalized to the number of BrdU^+ ^cells in sham-treated animals at various time points after systemic treatment with either BCNU or cisplatin. Data are means ± SEM. **(a,b) **Percent-corrected values of BrdU^+ ^cells after (a) BCNU treatment or (b) cisplatin treatment. Bars labeled with an asterisk show statistically significant (*P *< 0.01) differences from control animals. **(c) **Immunoperoxidase staining for detection of BrdU^+ ^cells in the lateral SVZ in representative sections from a 0.9% NaCl-injected control animal (C), one day (D1), and 42 days (D42) after systemic treatment with BCNU (3 × 10 mg/kg i.p.). **(d) **Diagrammatic representation of the part of the SVZ shown in (c) with adjacent part of the striatum (STR) and the overlying CC.

Analysis of DNA synthesis *in vivo*, as detected by BrdU labeling, revealed adverse effects of BCNU treatment in CNS regions in which cell proliferation in putative germinal zones is thought to be a critical component of normal tissue function (that is, the SVZ and the DG [58]), as well as in the CC (Figure 8a). BCNU treatment caused a reduction in the number of BrdU-incorporating cells for at least 6 weeks after the final (third) injection, with either no recovery or a continued fall in numbers of BrdU^+ ^cells to values 50–80% below control values. Thus, repetitive exposure to BCNU caused marked long-term impairments in cell proliferation in the CNS.

We combined *in vivo *labeling with BrdU with confocal analysis to determine whether BCNU preferentially reduced DNA synthesis in any particular cell population(s), and found that the distribution of BrdU incorporation between different cell populations was unchanged by the exposure to chemotherapy (Table 1). For example, in the CC, 86 ± 2% of the BrdU-labeled cells were positive for the Olig2 transcriptional regulator in control animals (and thus would be considered to be O-2A/OPCs [59-61]), and 86 ± 12% of the BrdU^+ ^cells were Olig2^+ ^in BCNU-treated animals. Similarly, the proportion of BrdU^+ ^cells that were DCX^+ ^(and thus would be considered to be neuronal precursor cells [44]) was unchanged in the SVZ (38 ± 5% in controls vs 43 ± 5% in treated animals) and in the DG (70 ± 6% in controls vs. 60 ± 14% in treated animals). Thus, the reduction in cell division associated with exposure to BCNU (as analyzed by BrdU incorporation) did not seem to specifically target any particular population of cells, at least when examined 1 day after completion of treatment.

**Table 1:** BCNU affects different neural progenitor cell populations equally *in vivo*

Cell population	SVZ control	SVZ BCNU	DG control	DG BCNU	CC control	CC BCNU
DCX^+^	38 ± 5	43 ± 5	70 ± 6	60 ± 14	ND	ND
Olig2^+^	14 ± 7	13 ± 13	15 ± 2	15 ± 3	86 ± 2	86 ± 11
S-100β^+^	2 ± 4	2 ± 3	10 ± 6	2 ± 3	10 ± 6	14 ± 11

In these experiments, BrdU-labeled cells were co-analyzed for expression of cell-type specific antigens by confocal microscopy, as in Figures 5–7, for the same animals as were analyzed for Figure 5. All cells were analyzed by *z*-stack analysis to confirm identity of the BrdU^+ ^incorporation and labeling with cell-type specific antibodies. Numbers are provided as average percentages ± SEM of all BrdU^+ ^cells identified for each animal, as described in Materials and methods. DCX expression was not analyzed (ND) in the corpus callosum (CC), because of the lack of neuronal progenitor cells in white-matter tracts. The data show that, despite the reduction in total numbers of BrdU^+ ^cells in each tissue, each individual cell population was affected similarly, and did not change in its proportional contribution to the entire population of BrdU^+ ^cells. The only possible exception to this is the representation of BrdU^+ ^GFAP^+ ^cells in the dentate gyrus (DG), but the difference between this set and controls did not achieve significance. SVZ, subventricular zone.

Treatment with three injections of cisplatin was also associated with reduced BrdU incorporation in the SVZ, DG, and CC when examined 1 day after the final injection (Figure 8b). In contrast to the effects of BCNU, however, the number of cells incorporating BrdU returned to normal levels in the DG and SVZ 6 weeks after treatment. Only in the CC was the number of BrdU^+ ^cells still reduced at this late time point.

### Cytarabine also exhibits preferential toxicity for CNS progenitor cells and oligodendrocytes, compromises cell division *in vitro*, and causes cell death and reduced cell division *in vivo*

To determine whether the effects seen so far were specific for DNA crosslinking agents, we extended our studies to include the antimetabolite cytarabine, which is commonly used in treating leukemia and lymphomas, and also has been associated with adverse neurological effects [25,62].

**Figure 9:**
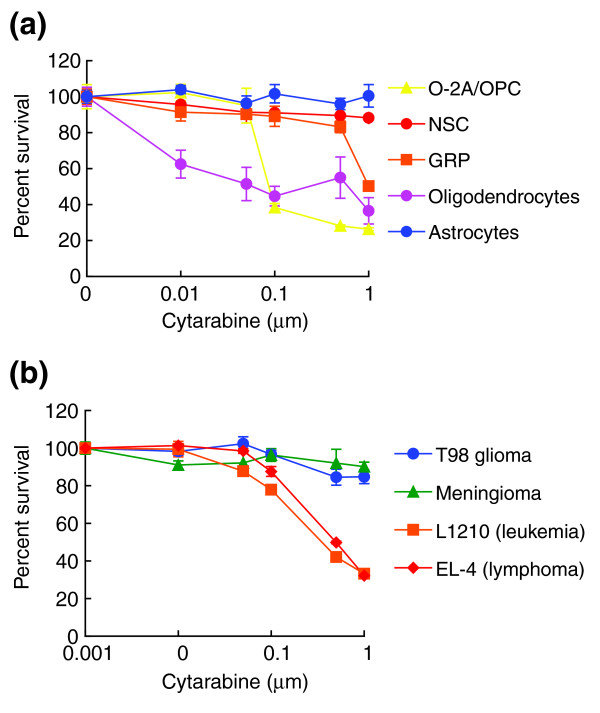
Primary CNS cells are equally or more vulnerable to cytarabine than cancer cells. Cells were plated on coverslips in 24-well plates at a density of 1,000 cells per well and allowed to grow for 24–48 h. On the basis of drug concentrations achieved in human patients, cells were exposed to cytarabine for 24 h. Cell survival and viability was determined after additional 24–48 h (see Materials and methods). **(a) **Rat neural cell types studied included O-2A/OPCs, oligodendrocytes, GRP cells, NSCs and astrocytes. **(b) **We also examined the T98 glioma cell line, a meningioma cell line, and the L1210 and EL-4 leukemia cell lines. To define the onset of cytarabine toxicity, cells were treated with cytarabine over a wide dose range (0.01–1 μM) extending downwards from the lower ranges achieved in high-dose therapy. Each experiment was carried out in quadruplicate and was repeated multiple times in independent experiments. Data represent mean of survival ± SEM, normalized to control values. There are no concentrations of cytarabine at which tumor cell lines were more sensitive O-2A/OPCs or oligodendrocytes.

Like cisplatin and BCNU, concentrations of cytarabine routinely achieved in the clinic were highly toxic for neural progenitor cells *in vitro*. Cerebrospinal fluid concentrations of cytarabine during conventional treatments are in the range of 0.1–0.3 μM, are ten times higher in high-dose applications, and can be 10,000 times higher following intrathecal application [63]. Exposure of primary neural cells to 0.1 μM cytarabine (equivalent to concentrations achieved in low-dose therapeutic utilization) for 24 hours killed more than 60% of O-2A/OPCs (Figure 9a). At this lower level of exposure, O-2A/OPCs were markedly more sensitive to the effects of cytarabine than were L1210 lymphocytic leukemia and EL-4 lymphoma cells – examples of tumor populations for which cytarabine would be used (Figure 9b). Exposure to 1 μM cytarabine (equivalent to the lower range of concentrations achieved in high-dose therapeutic applications, and an effective concentration for killing the L1210 and EL-4 cells) killed most O-2A/OPCs and around 50% of GRP cells.

Like cisplatin and BCNU, cytarabine toxicity was not limited to dividing cells, nor did it affect all dividing populations. Treatment for 24 hours with 0.1 μM of cytarabine induced a 2.4 ± 0.06-fold increase in the percentage of apoptotic TUNEL^+ ^oligodendrocytes, and treatment with 2 μM cytarabine for 24 hours killed 82.4 ± 5.8% of oligodendrocytes (data not shown). As these cells were not dividing in the culture conditions used, the toxicity of cytarabine also extends beyond division-dependent effects. Also as with cisplatin and BCNU, purified astrocytes and NSCs (which were dividing rapidly in the culture conditions used) were less sensitive to the effects of cytarabine, although even these populations were adversely affected by the millimolar concentrations (data not shown) achieved with intrathecal administration.

**Figure 10:**
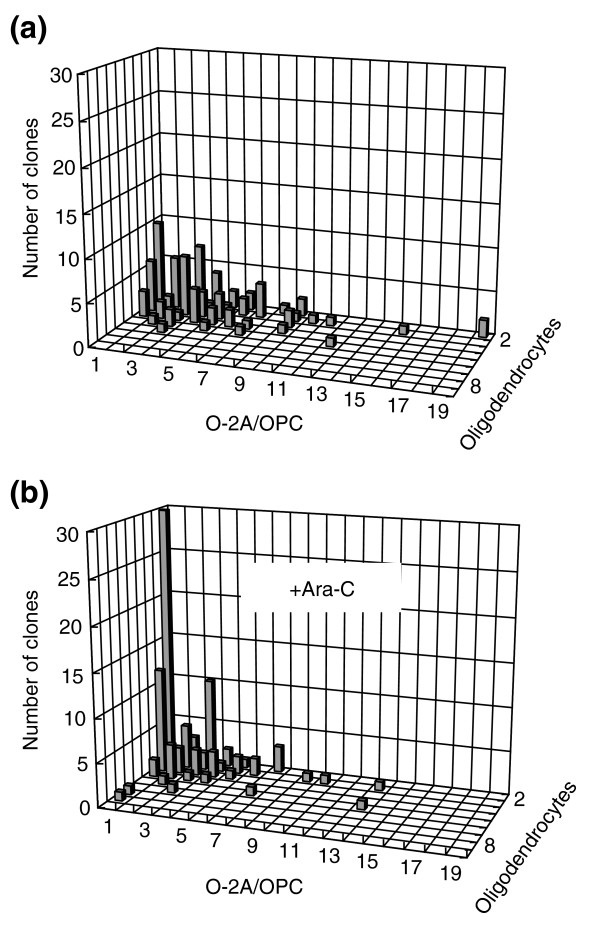
Low-dose cytarabine decreases division and promotes differentiation of O-2A/OPCs. Cells grown at clonal density were exposed 1 day after plating to low-dose cytarabine (0.01 μM for 24 h), a dosage that killed less than 5% of O-2A/OPCs in mass culture (Figure 9). The number of undifferentiated O-2A/OPCs and differentiated cells (oligodendrocytes) was determined in each individual clone from a total of 100 clones in each condition by morphological examination and by immunostaining with A2B5 and anti-GalC antibodies (to label O-2A/OPCs and oligodendrocytes, respectively), as in Figure 4. **(a) **Composition of progenitors and oligodendrocytes in a representative experiment of control cultures analyzed 6 days after plating optic nerve-derived O-2A/OPCs cells at clonal density. **(b) **In parallel cytarabine (Ara-C)-treated cultures analyzed 6 days after plating at clonal density (5 days after the start of cytarabine exposure), there was a marked increase in the representation of small clones consisting wholly of oligodendrocytes, a reduction in the representation of large clones, and a general shift of clone size towards smaller values. Experiments were performed in triplicate in at least two independent experiments.

As with BCNU and cisplatin, exposure to sub-lethal concentrations of cytarabine was associated with suppression of O-2A/OPC division in clonal assays. In these experiments, O-2A/OPCs were exposed to 0.01 μM cytarabine (a concentration equivalent to 10% or greater than that found in the cerebrospinal fluid in standard-dose applications of this chemotherapeutic agent [63]). Cytarabine was washed out after 24 hours, after which cultures were followed for 5 days. As shown in Figure 10, this transient exposure was not incompatible with continued division or survival, but was associated with a marked increase in the contribution of clones consisting of just one or two oligodendrocytes and no progenitor cells (with 16 out of 100 such clones seen in control cultures and 42 of 100 in those transiently exposed to 0.01 μM cytarabine). Moreover, there was also a reduction in the number of clones containing eight or more progenitors (with 13 of 100 such clones in control cultures and 4 out of 100 in cytarabine-treated cultures), along with a more general shift towards clones with fewer progenitor cells. Despite the adverse effects of even low-dose cytarabine on oligodendrocytes (Figure 9), transient exposure of O-2A/OPCs to cytarabine did not prevent the subsequent generation of oligodendrocytes, as shown in Figure 10.

**Figure 11:**
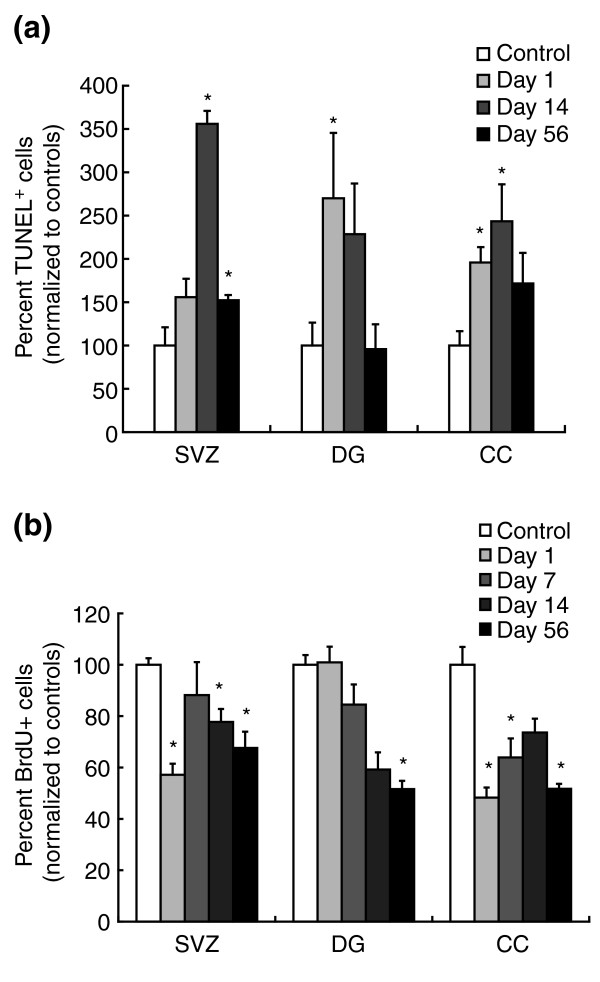
Systemic chemotherapy with cytarabine leads to increased and prolonged cell death, and decreased BrdU incorporation, in the adult mouse CNS. Cell death and BrdU incorporation were examined as in Figures 5 and 8. **(a) **The number of TUNEL^+ ^cells was analyzed in control animals and is presented as percent normalized values of controls. Each treatment group consisted of *n *= 5 animals, including control groups at each time point. Animals that received three cytarabine injections (250 mg/kg on days 1, 3, and 5 leading up to the analysis, where day 1 of analysis equals one day after the last treatment with cytarabine) show marked increases in cell death in the SVZ, CC and DG at various time points after treatment (*n *= 5 animals per group). **(b) **BrdU analysis. Animals were treated as for (a). As in Figure 8, the graphs show the percent BrdU^+ ^cells per brain area normalized to the number of BrdU^+ ^cells in sham-treated animals at various time points after systemic treatment with cytarabine. Data are means ± SEM; *P < 0.01 in comparisons with control animals.

Systemic treatment with cytarabine *in vivo *was associated with adverse effects on the CNS, in regard to both cell death and cell division (as indicated by BrdU incorporation). TUNEL staining was elevated in the SVZ, DG, and CC following treatment of mice with three injections of cytarabine (250 mg/kg, i.p., days 1, 3, and 5, as routinely used in mice [64]). Significantly greater numbers of TUNEL^+ ^cells were still found in the SVZ 56 days after the final treatment, and in the DG and CC on 1 and 14 days after treatment ended (Figure 11). The number of BrdU^+ ^cells was reduced in the SVZ at 1, 14, and 56 days after treatment, and was lower in the CC at all time points. DNA synthesis in the DG was significantly reduced below control levels only at the late time point of 56 days after the final cytarabine injection.

**Figure 12:**
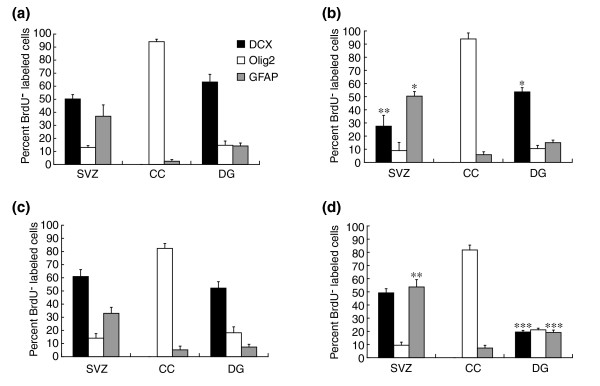
Co-analysis of BrdU incorporation with antigen expression indicates that division of both DCX^+ ^neuronal progenitors and Olig2^+ ^oligodendrocyte precursors is reduced by systemic exposure to cytarabine. In the CC, where there was an approximately 50% reduction in the number of BrdU^+ ^cells (see Figure 11b), the proportion of BrdU^+ ^cells that were Olig2^+ ^was no different between controls and treated animals on either day 1 (**(a) **control; **(b) **cytarabine) or on day 56 (**(c) **control; **(d) **cytarabine) after completion of treatment. Thus, the reduction in apparent division of Olig2^+ ^cells was proportionate to the overall reduction in all BrdU^+ ^cells. In contrast with effects on Olig2^+ ^populations in the corpus callosum, our analyses indicate an enhanced loss of DCX^+ ^cells from among the BrdU^+ ^population in both the SVZ and DG. This was particularly striking in the DG, where at 56 days post-treatment the proportion of BrdU^+ ^cells in the cytarabine-treated animals was < 40% of that seen in control animals. Data are means ± SEM; *P < 0.05, **P < 0.01, and ***P < 0.001 in comparisons with control animals.

**Figure 13:**
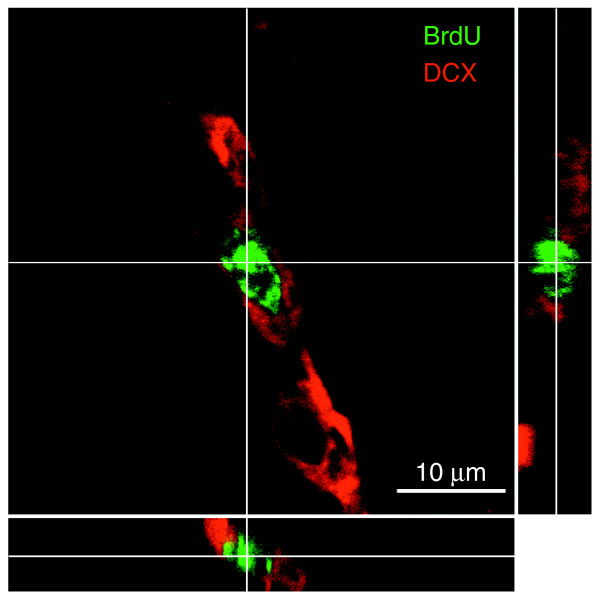
Slice (three-sided) reconstruction of a BrdU^+^/DCX^+ ^cell. Photographs were taken at 2 μm intervals. Identical analyses were conducted for every cell that was scored as BrdU^+ ^and expressing a cell-type specific antigen, as shown in Figure 4. The three-sided reconstruction shows that the BrdU^+ ^nucleus (green) belongs to the DCX^+ ^cell (red).

**Figure 14:**
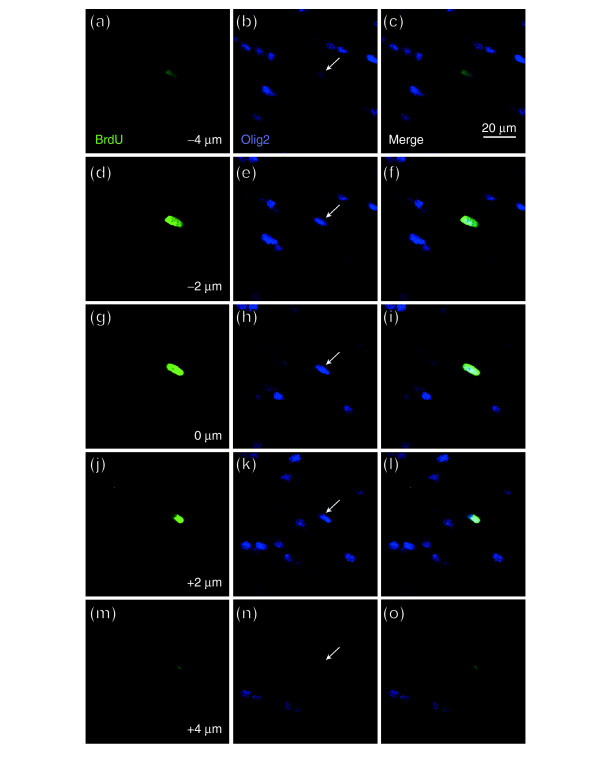
Representative *z*-stack of a BrdU^+^/Olig2^+ ^cell. Photographs were taken as for Figure 13. Identical analyses were conducted for every cell that was scored as BrdU^+ ^and expressing a cell-type specific antigen, as shown in Figure 4. As seen, the BrdU^+ ^nucleus (green) was that of the Olig2^+ ^cell (blue) indicated by a white arrow. Each row shows, from left to right, BrdU incorporation, staining for Oligo2, and the merged image. Images taken at **(a-c) **-4 μm; **(d-f) **-2 μm; **(g-i) **0 μm; **(j-l) **2 μm; **(m-o) **4 μm.

Examining the effects of cytarabine on different cell populations, we found that both neuronal precursors [44] and oligodendrocyte precursors were affected *in vivo*. In the CC, where there was around 50% reduction in the number of BrdU^+ ^cells observed in tissue sections from animals sacrificed 1 day after the completion of treatment, the proportion of BrdU^+ ^cells that were Olig2^+ ^(that is, were oligodendrocyte precursor cells [59-61]) was no different between controls and treated animals (Figures 12, 13, 14). This result held true also at day 56, when the proportionate representation of Olig2^+ ^cells among the BrdU^+ ^population was unchanged both in untreated and treated animals, despite a continued 50% reduction in the total number of BrdU^+ ^cells observed.

In contrast with effects on Olig2^+^/BrdU^+ ^populations in the CC, our analyses raise the possibility of a somewhat enhanced loss of DCX^+ ^cells from among the BrdU^+ ^population in both the SVZ and DG (Figure 12). In the SVZ, at 1 day after treatment, there was a disproportionate and significant reduction in the percentage of DCX^+^/BrdU^+ ^cells, which represented 50 ± 3% of the cells incorporating BrdU in control animals and only 28 ± 8% in animals treated three times with cytarabine. This disproportionate reduction in the percentage of BrdU^+ ^cells that was DCX^+ ^did not seem, however, to be maintained over time in the SVZ, and at day 56 the proportion of BrdU^+ ^cells that were DCX^+ ^was not different in controls versus treated animals. In contrast, in the DG, a reduction in representation of DCX^+ ^cells was also seen, except that in this case there was a marked 60% reduction in the proportion of BrdU^+ ^cells that were DCX^+ ^when day 56 results were compared with either controls of the same age or the proportionate representation of this population at day 1 after injury.

In the SVZ and the DG, cytarabine application was also associated with an increased representation of GFAP^+ ^cells among the BrdU-incorporating populations. This increased representation of GFAP^+ ^cells was seen at both day 1 and day 56 in the SVZ and on day 56 in the DG. In addition, BrdU^+ ^cells that did not label with any of the cell-type-specific antibodies used in these studies were more prominent in treated animals than in controls at day 1 (but not at day 56) in the SVZ, and were found in the DG at both time points (data not shown). The only tissue in which these unlabeled cells provided a greater than 10% contribution to the entire BrdU^+ ^population was in the DG. Such cells represented around 2% and around 20% of the BrdU-labeled cells at days 1 and 56, respectively, of all BrdU-labeled cells in control animals versus around 15% and about 35% (for days 1 and 56, respectively) of the entire BrdU^+ ^population in the treated animals.

## Discussion

We found that normal neural progenitor cells and oligodendrocytes of the CNS are exceptionally vulnerable to the toxic effects of the chemotherapeutic agents BCNU, cisplatin, and cytarabine. Vulnerability to these drugs was observed for all classes of lineage-restricted progenitor cells that can be readily grown as purified cell populations. Moreover, vulnerability was not restricted to dividing cells, as nondividing oligodendrocytes were also targets of these drugs, at exposure levels routinely achieved during treatment. *In vitro *analyses of purified cell populations were highly predictive of effects seen following systemic treatment with any of the chemotherapeutic agents *in vivo*. Comparative analysis of multiple cancer cell lines from different tissues only identified one cell line in which vulnerability was comparable to that observed for primary neural progenitor cells, with most such cell lines being more resistant to these agents than the normal cells (despite often being chosen because of their previous use in studies on the response to the drugs studied). Thus, it appears that the vulnerability of multiple normal cell populations of the CNS to cisplatin, BCNU, and cytarabine rivals the vulnerability of cancer cells themselves. The fact that toxicities for neuronal precursors, glial precursors, and oligodendrocytes, and toxicity in three different regions of the CNS, are associated with systemic application of chemotherapeutic drugs is of particular concern, as such toxicity would be applicable to treatment of all forms of cancer. Moreover, our studies demonstrate that the adverse effects of systemic application are not limited to the classes of DNA cross-linking agents represented by BCNU and cisplatin, but also are observed with the antimetabolite cytarabine. Thus, the adverse effects observed in the present studies may be relevant in understanding the side effects of multiple classes of chemotherapeutic drugs.

This is the first study of which we are aware that demonstrates that neural progenitor cells and oligodendrocytes are exceptionally vulnerable to the action of chemotherapeutic drugs *in vitro *and *in vivo*, even when applied extra-cranially. This study also suggests that, at least in the CNS, it is progenitor cells and not stem cells that are the most vulnerable targets. Adverse effects are known to occur clinically with all the agents we studied, both acutely and as delayed neurotoxicities (such as cognitive impairment) that may only become apparent years after treatment. For example, BCNU treatment has been associated with significant changes in mental status and with white matter degeneration [23,24]. Cisplatin at high doses has been associated with leukoencephalopathy and destruction of CNS white matter [25]. Application of cytarabine, the third drug examined in our studies, has also been associated with acute encephalopathy, confusion, memory loss, and white matter changes [25,62]. The vulnerability of neural progenitor cells and oligodendrocytes to these drugs, which was also observed in our antigenic analysis of TUNEL-labeled cells in the CNS of animals exposed to BCNU *in vivo *and BrdU-labeled cells exposed to either BCNU or cytarabine, may provide an explanation for the neurotoxic consequences of the treatments and also may be relevant to understanding long-term toxicities. It was particularly striking that O-A/OPCs and oligodendrocytes were one to two orders of magnitude more sensitive to cisplatin or cytarabine than has previously been observed in studies on multiple neuronal populations from both the CNS and the peripheral nervous system [65-70].

The toxicities seen in our studies occurred well within the concentration ranges achieved for these agents in CSF during cancer therapy. For example, cisplatin toxicity for multiple neural cell types was observed at concentrations as low as 0.1 mM (Figure 3). CSF concentrations for cisplatin in conventional or low-dose intravenous applications are between 0.6 and 2.8 μM [33] but can reach up to 80 μM in high-dose applications [36]. Moreover, much higher concentrations in brain tissue and CSF have been reported after intra-arterial applications, liposomal encapsulations, after previous radiation, or in cases of blood-brain barrier disruption [37,38]. BCNU toxicity was observed at concentrations as low as 5 μM. This agent is highly lipophilic, and about 80% of plasma levels are detectable in CSF and brain tissue [34]. CSF concentrations of BCNU are in the range of 8–10 μM after intravenous applications [35], but can be 100–1,000-fold higher after local applications via biodegradable polymer wafers [39,40]. Similarly, the toxicity of cytarabine was already apparent at concentrations as low as 0.1 μM or less, compared with CSF concentrations during conventional treatments in the range 0.1–0.3 μM, and concentrations that are ten times higher in high-dose applications, and 10,000 times higher following intrathecal application [63].

*In vitro *studies further indicated that the toxicity of BCNU, cisplatin, and cytarabine is not limited to the induction of cell death, but is also associated with the suppression of cell division of O-2A/OPCs even when applied transiently at levels that cause little or no cell death, and that represent small fractions of the CSF concentrations achieved with systemic chemotherapy. The suppression of division was particularly striking in that a single transient exposure of dividing O-2A/OPCs to BCNU, cisplatin, or cytarabine was sufficient to cause a marked reduction in subsequent cell division at the clonal level. Such a loss of dividing cells would compromise the ability of dividing progenitor cells to contribute to repair processes, and could also contribute to long-term or delayed toxicity reactions.

The observations that BCNU, cisplatin, and cytarabine all cause dividing O-2A/OPCs to undergo a greater extent of oligodendrocyte generation are as predicted from our studies on the role of intracellular redox state in controlling the balance between self-renewal and differentiation [41], and from observations that all three agents cause cells to become more oxidized [69,71-75]. In our studies on redox regulation of precursor cell function, we found that O-2A/OPCs that are slightly (around 20%) more oxidized have a higher probability of undergoing differentiation, whether this oxidative status is due to cell-intrinsic mechanisms, exposure to pharmacological pro-oxidants or to physiological inducers of oligodendrocyte generation (such as thyroid hormone) [41,43]. Even when this shift in differentiation probability is relatively small [76], cumulative effects over multiple cell generations can lead to differentiation outcomes in which clonal composition is clearly different but in which analysis at delayed time points is required for the reduction in progenitor cell representation to translate into markedly smaller clonal sizes.

*In vitro *studies on purified cell populations appeared to accurately predict sensitivities observed *in vivo*. Combined analysis of TUNEL and antigen expression demonstrated death of both neuronal and glial precursors, as well as of oligodendrocytes. Combined analysis of BrdU labeling and antigen expression similarly revealed reductions in BrdU incorporation in neuronal precursors of the hippocampus and in glial precursor cells of the CC. The high level of correlation between *in vitro *and *in vivo *outcomes suggests that purified populations of the cell types studied can provide a means of rapidly analyzing other cancer therapies.

Although all chemotherapeutic drugs examined were associated with toxicity *in vivo*, there were important differences between them. BCNU was associated with particularly severe and prolonged cell death *in vivo*, while cell death induced by cisplatin was less severe and eventually returned to normal values. Cytarabine was associated with increased cell death for at least 14 days after treatment ended, with values tending towards or at base-line levels of TUNEL labeling at 56 days post-treatment. Whether the less severe effects of cisplatin in this regard were due to different drug characteristics in terms of blood-brain barrier permeability is not known (although, in this regard, it should be noted that cisplatin application *in vivo *may actually cause opening of this barrier [77]).

All three agents examined were associated, moreover, with continued reductions in cell division in one or more CNS regions after treatment ended, suggesting a long-lasting depletion of populations required for cell replenishment. Nonetheless, the fact that some BrdU-incorporating cells remained in all brain regions examined raises the question of whether treatments analogous to those used to enhance bone-marrow function after cancer treatment may be applicable some day to enhancing the function of the normal dividing cells of the CNS during or after cancer treatment, possibly even using the same cytokines that are used to enhance cell repopulation from the bone marrow [78-80].

The effects of cytarabine on the different cell populations that incorporated BrdU *in vivo *were particularly surprising in the context of previous observations that cytarabine exposure *in vivo *(delivered by infusion onto the cortex for 7 days) is associated with a repopulation of the SVZ after treatment ceases [21,81]. In contrast, our own studies indicate that this repopulation of dividing cells does not occur in the CC or DG, and may not endure in the SVZ (Figure 11). Although previous studies differ from our own in delivery methods and dosages applied, it may also be that the capacity for repopulation of dividing cells differs in different regions of the CNS. Moreover, it may be that the repopulation of the dividing cells of the SVZ is a transient phenomenon, as the latest time point examined in our studies was associated with a fall in the levels of BrdU incorporation to levels seen 1 day after treatment ended.

Taken together with recent studies on the effects of irradiation on the CNS [82], our results indicate that damage to CNS progenitor cells is an apparent correlate of both the main treatments for cancer. Monje *et al*. [82] suggested that the adverse effects of irradiation on the hippocampus might be causally related to the neurological symptoms and cognitive decline associated with this treatment. This suggestion would also apply to the effects of chemotherapy.

There are many ways in which the effects of chemotherapy may be even more of a concern than the effects of irradiation, beginning with the fact that whereas radiation damage is caused by therapy targeted to the CNS, toxicity after chemotherapy also occurs after systemic administration of these compounds. Moreover, our studies also reveal that the range of CNS cell types vulnerable to the effects of chemotherapy is greater than has been studied for irradiation, and demonstrate toxicity of chemotherapeutic agents for glial progenitor cells and for oligodendrocytes, as well as for the hippocampal precursor cells that have been examined in studies on the effects of irradiation [82]. Yet another difference between the effects of these two modes of treatment is that irradiation-associated impairment of neurogenesis appears to be a secondary effect of inflammation, and can thus be reduced with anti-inflammatory agents [83]. In contrast, our preliminary analyses of chemotherapy-treated animals have not revealed any increased microglial activation, a hallmark of CNS inflammation (J. D. and M. N., unpublished work). Thus, there is presently no reason to think the adverse effects of chemotherapy might be ameliorated by control of inflammation. The two sets of studies also differed in severity of outcome, in that our study reveals a partial fall in the representation of DCX^+ ^neuronal precursor cells whereas the studies on irradiation revealed a virtually complete lack of neurogenesis [82]. While it will be of interest to extend examination of both treatment paradigms, it is nonetheless the case that both studies raise the concern that neurogenesis in the brain is vulnerable to both forms of cancer treatment.

Our studies have multiple implications for future strategies of cancer treatment. As doses of BCNU, cisplatin, and cytarabine that killed even chemosensitive cancer cell lines were equally or more toxic for neural progenitor cells and oligodendrocytes, it seems that any concentration of these chemotherapeutic agents sufficient to harm cancer cells may also damage many cell populations of the CNS. That cisplatin may have less severe long-term effects than BCNU might be construed as encouragement that less toxic treatments can be developed with existing chemotherapeutic agents. It is also possible, however, that our results actually understate the extent of damage that occurs in association with chemotherapy. Such treatment is typically applied for several courses over an extended period of time. Furthermore, current treatment protocols simultaneously apply multiple different chemotherapeutic agents. This issue is of particular concern in the light of reports that agents such as cisplatin or BCNU can cause opening of the blood-brain barrier [77,84], which could allow entry of adjunctive non-lipophilic agents into the CNS. In addition, there are multiple therapeutic regimes associated with higher concentrations of drugs than those we have studied (for example, intra-arterial administration, liposome-encapsulated drugs, or locally applied biodegradable wafers in the treatment of brain tumors). Moreover, the advances that have been made in rescuing patients from the toxicity of chemotherapeutic agents for bone marrow have been associated with a tendency to apply yet higher doses of these agents, thus potentially increasing the risk of neurotoxicity.

As chemotherapy will remain a cornerstone of cancer therapy for the foreseeable future, the potential ramifications of this work for present and future cancer treatments seem clear. Plainly, it is of great importance to identify the neural populations at risk during any cancer treatment in order to develop means of reducing neurotoxicity and preserving the quality of life in long-term survivors. This is an issue of great concern, particularly in the light of recent studies favoring the use of more aggressive and high-dose regimens or of newer drugs that target receptor tyrosine kinase signaling pathways that are critical regulators of neural progenitor and stem-cell function. In this context, it will be of particular importance to include more profound analysis of CNS toxicity in the assessment of new candidate chemotherapeutic drugs, an evaluation that currently is not consistently performed. It will also be critical to understand why some patients have adverse side effects (whether neurological or non-neurological), whereas others are spared such damage, and to determine the value of low-dose (metronomic) therapies [85] in avoiding damage to the CNS without compromising treatment outcome. In this regard, it is of concern that our *in vitro *results raise the possibility that even exposure to very low levels of these agents may compromise progenitor cell division. It is clearly vital to identify therapeutic approaches that do not share these problems, either by enabling targeted killing of cancer cells or through selective protection of normal cells during cancer treatment. The strong correlations between our *in vitro *and *in vivo *analyses indicate that the same approaches we used to identify the reported toxicities can also provide rapid *in vitro *screens for analyzing new therapies and discovering means of achieving selective protection or targeted killing. In light of the ease of use of these *in vitro *and *in vivo *assays, applying them early in the drug-discovery process may enable a more rapid identification of treatments able to eliminate cancer cells without compromising the patient's quality of life.

## Materials and methods

### Preparation of primary cell cultures

*In vitro *studies were performed on purified cultures of primary CNS cells. Multipotent neuroepithelial cell cultures were prepared from embryonic day 10.5 (E10.5) Sprague-Dawley rat spinal cord, as previously described [29,86]. NRP cells were prepared by inducing neuronal differentiation from multipotent NEP cells, as described [29]. Glial-restricted precursor cells (A2B5^+ ^GRP) were isolated directly from E13.5 Sprague-Dawley rat spinal cord [30]. Purified O-2A/OPCs were prepared from the CC or optic nerve of 7-day-old Sprague-Dawley rats using a specific antibody capture assay [42]. Purified oligodendrocytes were generated from O-2A/OPC cell cultures by growing cells in presence of thyroid hormone (45 μM) to induce oligodendrocyte differentiation [42]. Purified cortical astrocytes were prepared from 1- to 2-day-old Sprague-Dawley rats as described [87]. Multipotent and lineage committed human embryonic neural progenitor cells were obtained from Clonetics (San Diego, CA, USA) and propagated as described previously [32,88].

### Glioma cells and other cancer cell lines

Brain tumor cells used in this study were isolated from patients with glioblastoma multiforme (1789, UT-12, UT-4 and T98 cell lines). Brain tumor cells were grown in serum-free conditions in 50% chemically defined medium (DMEM/F-12, supplemented with PDGF-AA and basic fibroblast growth factor (FGF) at 10 ng/ml each) and 50% astrocyte-conditioned medium (derived from cortical astrocyte cultures [87]). In addition, SW480 and HT-29 colon carcinoma cells, uterine (MES), breast (MCF-7 and MDA-MB-231), and ovarian cancer (ES-2) cells, L1210 lymphocytic leukemia and EL-4 lymphoma cells and a meningioma cell line, derived from a patient with a meningotheliomatous meningioma, were also evaluated. These cells were propagated in DMEM/F-12 in presence of 5% FCS, except for EL4 and L1210 (DMEM + 10% horse serum) and ES-2 (McCoy's 5A (Cellgro) + 10% FCS). Sensitivity of cancer cells to chemotherapeutic agents showed no significant differences whether cells were assayed in the presence or absence of serum.

### *In vitro *toxicity and viability assay

For *in vitro *toxicity studies, cells were plated on coverslips at a density of 1,000 cells per well. After 24–48 h, cells were exposed to increasing drug concentrations of BCNU (5–200 μM) for 1 h, cisplatin (0.1–100 μM) for 20 h or cytarabine (0.01 μM to 2 μM) for 24 h. Cells were then allowed to recover for 24–48 h, the times being based on clinically applied dosages and elimination half-times of these drugs *in vivo*. Cell survival and viability was determined using the 3,(4,5-dimethylthiazol-2-yl) 2,5-diphenyl-tetrazolium-bromide (MTT) assay in combination with 4',6-diamidino-2-phenylindole (DAPI) staining to visualize DNA. The MTT assay was performed as described and also combined with immunofluorescence [89]. This assay is more sensitive than the plate reader assay used in our previous studies on the effects of BCNU on oligodendrocytes, O-2A/OPCs, and astrocytes [22]. After MTT and DAPI staining, surviving cells were determined by microscopically counting all individual cells in control and treatment groups. All counting was done blinded by a separate investigator. Each experiment was carried out in quadruplicate and was repeated at least twice in independent experiments. Data points represent mean from single experiments and error bars shown in figures represent ± standard error of the mean (SEM).

### Immunocytochemistry and immunofluorescence staining *in vitro*

Cell cultures were immunostained as described [29-32,41], using the following antibodies: A2B5 mouse IgM mono-clonal antibody (mAb) (Developmental Hybridoma Bank, Iowa City, IA, USA); anti-galactocerebroside mouse IgG_3 _(GalC, 1:1, Developmental Hybridoma Bank, 1:50); anti-GFAP polyclonal rabbit Ig (DAKO, Copenhagen, Denmark, 1:400); anti-neurofilament protein mouse mAb IgG_1 _(NF-L, Chemicon, Temecula, CA, USA, 1:200), and anti-b-III-tubulin mouse mAb IgG_2b _(Biogenex, San Ramon, CA, 1:400). Antibody binding was detected with appropriate fluorescent dye-conjugated secondary antibodies (10 mg/ml, Southern Biotechnology), or Alexa fluorophore-coupled antibodies at a concentration of 1 μg/ml (Molecular Probes, Eugene, OR, USA).

### Chemotherapy application *in vivo*

For *in vivo *experiments, CBA mice at 6–8 weeks of age were treated with chemotherapy under approved protocols. BCNU, cisplatin, or cytarabine were administered via i.p. injections. Animals received BCNU, cisplatin, or cytarabine as three consecutive injections (3 × 10 mg/kg, 5 mg/kg, or 250 mg/kg body weight, respectively). Control animals received equal amounts of 0.9% NaCl i.p. Animals were sacrificed on days 1, 10, and 42 after completion of treatment for cisplatin and BCNU (where day 0 equals the time of the very last injection of the agent). For all *in vivo *experiments, animals were perfused transcardially with 4% paraformaldehyde in phosphate buffer (pH 7.4), under deep anesthesia using Avertin (tribromoethanol; Sigma, St Louis, MO, USA; 250 mg/kg, 1.2% solution).

### Immunofluorescence and TUNEL staining *in vivo*

Free-floating sections (40 μm) were used for all *in vivo *experiments for TUNEL staining and combined immunofluorescence staining. Detection of nuclear profiles with DNA fragmentation, one of the hallmarks of apoptosis, was performed using a TUNEL assay on free-floating brain sections based on the ApopTag-In-Situ Cell-Death-Detection Kit (Intergene, Purchase, NY, USA), according to the manufacturer's recommendations. The TUNEL assay was followed by DAPI counterstaining to visualize nuclear profiles in all *in vitro *assays and when sections were analyzed in a fluorescence microscope.

Briefly, sections were rinsed in TBS (0.9% NaCl and 0.1 M Tris-HCl pH 7.5) for 10 min, then exposed to a series of increasing concentrations of ethanol (50%, 70%, and 90% for 2 min each), followed by a 10 min incubation in 100% ethanol and a decreasing series of ethanol in 90%, 70%, and 50% for 1 min each, followed by rinsing in distilled water. After three rinses in TBS, the sections were exposed to equilibration buffer for 1 min at room temperature, and reaction buffer (TdT solution) for 1 h at 37°C, as per the manufacturer's recommendations. The reaction was terminated using the Stop buffer for 10 min at room temperature. Sections were rinsed 3× in TBS and 1× in TBS^+ ^(TBS/0.1% Triton X-100/3% donkey serum) for 1 h to reduce background staining. Fragmented DNA was detected by incubation of sections in an anti-digoxigenin-FITC (fluorescein isothiocyanate) antibody for 1 h at 4°C.

To combine TUNEL staining with immunofluorescence staining for different cell-lineage markers, TUNEL staining was carried out first, followed by exposure with either one of the following primary antibodies for 24 to 48 h: mouse anti-NeuN (1:500, Chemicon), mouse anti-DXC (double-cortin) (1:500, Santa Cruz, Santa Cruz, CA, USA), rabbit anti-active caspase-3 (1:1000, R&D systems, Minneapolis, MN, USA), rat anti-S-100b (1:2500, Swant, Bellinzona, Switzerland), rabbit anti-NG2 (1:2000, gift of William Stallcup, Burnham Institute, La Jolla, CA, USA), mouse anti-MBP (1:1000, Chemicon), mouse anti-CNPase (1:2000, Sigma) and rabbit anti-GFAP (1:2500, DAKO). All secondary antibodies, generated in donkey (anti-rat, anti-rabbit, and anti-mouse), were coupled to TritC, FitC or Cy5 (Jackson Immuno Research, West Grove, PA, USA) for *in vivo *staining and were used according to the species of primary antibody. Free-floating sections were incubated with secondary antibodies for 4 h in TBS^+^. All secondary antibodies were used at a concentration of 1:500. After several washes in TBS, sections were mounted on gelatin-coated glass slides using Prolong Antifade mounting medium (Molecular Probes).

Fluorescent signals were detected using a confocal laser-scanning microscope Leica TCS SP2 (Heidelberg, Germany) and a 40× oil-immersion lens. All fluorescent images were generated using sequential laser scanning with only the corresponding single wavelength laser line (488 nm, 568 nm, and 647 nm, for each fluorescent channel, respectively), activated using acousto-optical tunable filters to avoid cross-detection of either one of the fluorescence channels. In addition, pinhole settings corresponding to an optical thickness of less than 2 mm were used to avoid false-positive signals from adjacent cells.

### BrdU incorporation assay, BrdU labeling, and immunoperoxidase staining for BrdU detection

To label the proportion of dividing cells engaged in DNA synthesis *in vivo*, mice received a single injection of 5-bromodeoxyuridine (50 mg/kg body weight), dissolved in 0.9% NaCl, filtered at 0.2μm, and applied i.p. 4 h before perfusion. Free-floating sections were treated with 0.6% H_2_O_2 _in TBS (0.9% NaCl and 0.1 M Tris-HCl pH 7.5) for 30 min to block endogenous peroxidase. For DNA denaturation, sections were incubated for 2 h in 50% formamide/2× SSC (0.3 M NaCl and 0.03 M sodium citrate) at 65°C, rinsed for 5 min in 2× SSC, incubated for 30 min in 2 N HCl at 37°C, and rinsed for 10 min in 0.1 M boric acid pH 8.5. Several rinses in TBS were followed by incubation in TBS/0.1% Triton X-100/3% donkey serum (TBS^+^) for 30 min and incubation with rat anti-BrdU antibody (Harlan Sera Lab, Loughborough, UK, 1:2500) in TBS^+ ^overnight at 4°C. Sections were rinsed in TBS^+ ^and incubated for 1 h with biotinylated donkey anti-rat antibody. Sections were rinsed several times in TBS and avidin-biotin-peroxidase complex (ABC system, Vector Laboratories, Burlingame, CA, USA) was applied for 1 h, followed by peroxidase detection for 5 min (0.25 mg/ml DAB, 0.01% H_2_O_2_, 0.04% NaCl). After several washes in TBS, sections were mounted on gelatin-coated glass slides using Prolong Antifade mounting medium (Molecular Probes).

To analyze BrdU incorporation in specific cell types, anti-BrdU immunostaining was combined with immunolabeling to identify DCX^+ ^neuronal precursor cells, Olig2^+ ^oligodendrocyte precursor cells (defined as cells that were BrdU^+ ^and Olig2^+^, in order to discriminate these cells from Olig2^+ ^nondividing oligodendrocytes [59-61]), and GFAP^+ ^cells (which would have been astrocytes in the CC or DG or, in the SVZ, may also have been stem cells). Labeling and confocal analysis was carried out as for the combination of immunolabeling with TUNEL staining. A minimum of 50 BrdU^+ ^cells were counted for each labeling condition in each animal (*n *= 3 animals in each group examined), with the sole exception of the DG of the animals examined 56 days after cytarabine treatment (for which an identical number of sections were examined as in control animals, but the frequency of labeled cells was not sufficient to reveal 50 cells in these sections). Rabbit anti-Olig2 antibody was a kind gift from David Rowitch.

### Histology

Brains were cut coronally as 40-μm sections with a sliding microtome (Leica, SM/2000R) and stored at -20°C in a cryoprotectant solution (glycerol, ethylene glycol, and 0.1 M phosphate buffer pH 7.4, 3:3:4 by volume). Quantification of BrdU^+ ^cells was accomplished with unbiased counting methods. BrdU-immunoreactive nuclei were counted in one focal plane to avoid oversampling. Brain structures were sampled either by selecting predetermined areas on each section (lateral subventricular zone = SVZ) or by analyzing the entire structure on each section (CC, DG of the hippocampus). Differences were considered significant when *p *< 0.01. SVZ: BrdU^+ ^cells were counted in every sixth section (40 μm) from a coronal series between interaural anterior-posterior (AP) +5.2 mm and AP +3.9 mm (the anterior commissure crossing). BrdU^+ ^cells were counted along the lateral ventricular wall up to 200 mm from the lateral ventricle wall. Corpus callosum (CC): BrdU^+ ^cells were counted in every sixth section (40 μm) from a coronal series between interaural AP +5.2 mm and AP +3.0 mm in the entire extension of the rostral and medial part of the CC and analyzed as for SVZ. Dentate gyrus (DG) of hippocampus: BrdU^+ ^cells were counted in every sixth section (40 μm) from a coronal series between interaural AP +2.5 mm and AP +1.1 mm. BrdU^+ ^cells were counted in the area of the DG, including the hilus, subgranular zone (SGZ), and the granule cell layer (GCL) and analyzed as for SVZ. Quantitative data in all figures is presented as mean percentage normalized to control animals. Error bars represent ± SEM.

### Images and data processing and statistics

Digital images were captured using a Nikon Eclipse E400 upright microscope with a spot camera (Diagnostic Instruments, Sterling Heights, MI, USA) and the spot advanced software for Macintosh (Diagnostic Instruments), or using the confocal laser-scanning microscope (Leica TCS SP2). Photomicrographs were processed on a Macintosh G4 and assembled with Adobe Photoshop 7.0 (Adobe Systems, Mountainview, CA, USA). In all comparisons, unpaired, two-tailed Student's t-tests were used.
